# Empathy and aggressive behavior from teenagers in educative institutions in Monteria, Colombia

**DOI:** 10.1192/j.eurpsy.2022.221

**Published:** 2022-09-01

**Authors:** E.P. Ruiz Gonzalez, F. Delgado Sanchez, M. Muñoz Argel, P. Grasso Imig, M. García Castañeda

**Affiliations:** 1 Universidad Pontificia Bolivariana, Cordoba, Monteria, Colombia; 2 Universidad Abierta Interamericana, Ciudad Autónoma De Buenos Aires, Buenos Aires, Argentina

**Keywords:** aggressive, Empathy, Adolescents

## Abstract

**Introduction:**

According to the World Health Organization (2016), adolescence is one of the most important transitional steps in the life of a human being, recognized by an accelerated rate of growth and changes in behavior. Adolescents from Colombia have reached this step, immersed in a context with a history of social, interpersonal and economic violence. In this sense, study of constructs such as empathy and aggressive behaviors are crucial to appease a healthy school coexistence and thus, contribute to a peace cultur

**Objectives:**

Analyze the relationship between empathy and aggressive levels from adolescents.

**Methods:**

This study was done through a cross-sectional study of correlational scope in 240 (N= 240) students. The Prosocial Behavior Questionnaire developed by Martorell and Gonzalez (1922) and the Aggressive questionnaire, developed by Buss and Perry (1992) were applied. The first one was used to measure empathy and the latter to appraise aggressiveness.

**Results:**

There was evidenced of adequate levels of empathy and a great percentage of medium levels of verbal and physical aggressiveness. (Graph 1). In addition, there was a significant statistical correlation of negative magnitude between these variables (Table 1).

**Figure:**
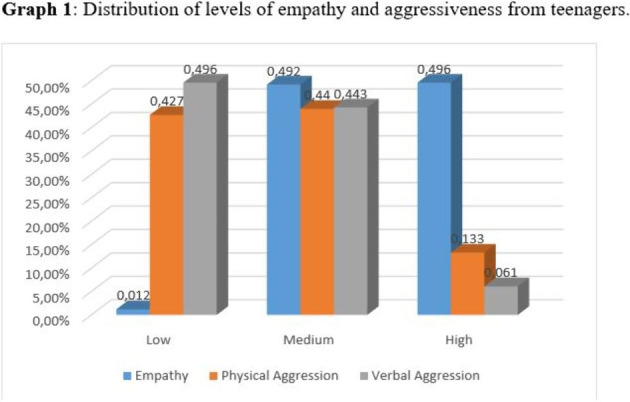

**Figure:**
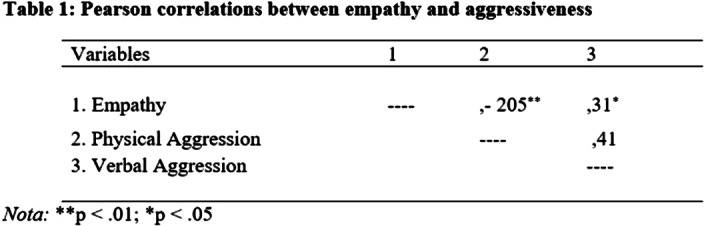

**Conclusions:**

It was concluded that the higher the optimal levels of empathy, the lower the aggressive behavior presented by teenagers.

**Disclosure:**

No significant relationships.

